# Isotropic, high-resolution, whole-chest inversion recovery contrast-enhanced magnetic resonance angiography in under 4.5 min using image-based navigator fluoro trigger

**DOI:** 10.3389/fcvm.2025.1549275

**Published:** 2025-04-30

**Authors:** Jason Craft, Roosha Parikh, Josh Y. Cheng, Nancy Diaz, Karl P. Kunze, Michaela Schmidt, Radhouene Neji, Amanda Leung, Suzanne Weber, Jonathan Weber, Timothy Carter, Sylvia Biso, Ann-Marie Yamashita, Eric H. Wolff, Claudia Prieto, Rene M. Botnar

**Affiliations:** ^1^ DeMatteis Cardiovascular Institute, St. Francis Hospital & Heart Center, Roslyn, NY, United States; ^2^ MR Research Collaborations, Siemens Healthcare Limited, Camberley, United Kingdom; ^3^ Siemens Healthineers, Erlangen, Germany; ^4^ School of Biomedical Engineering and Imaging Sciences, King’s College London, London, United Kingdom; ^5^ Department of Cardiothoracic Surgery, Good Samaritan University Hospital, West Islip, NY, United States; ^6^ School of Engineering, Pontificia Universidad Católica de Chile, Santiago, Chile; ^7^ Institute for Biological and Medical Engineering, Pontificia Universidad Católica de Chile, Santiago, Chile

**Keywords:** magnetic resonance angiography, motion correction, image-based navigator, time resolved angiography, variable density sampling

## Abstract

**Background:**

Serial assessment of the thoracic aorta with magnetic resonance angiography (MRA) is desirable due to 3D volumetric dataset, high spatial resolution, and lack of ionizing radiation. Electrocardiogram (ECG) gated, contrast-enhanced (CE), inversion recovery gradient echo MRA is efficient and historically provides low artifact burden, but the window for imaging with weak albumin binding extracellular gadolinium based contrast agents is small. Our purpose was to acquire whole-chest gated CE-MRA with 1.2 mm^3^ resolution using image-based navigator (iNAV) for motion correction/contrast monitoring, and variable density sampling in 4–5 min. Image quality and vessel diameter reproducibility are assessed against time resolved MRA (TR-MRA).

**Methods:**

iNAV CE-MRA and TR-MRA were obtained prospectively in 40 patients and reviewed by 3 blinded cardiologists for vessel diameter and image quality rated on a four point scale: (1) non-diagnostic; (2) poor-significant blurring; (3) good-mild blurring; and (4) excellent. Reproducibility and image quality were evaluated using the concordance correlation statistic and Cohen's kappa with mean differences evaluated using paired *t*-tests and repeat-measures ANOVA.

**Results:**

iNAV CE-MRA scan time was 4.2 ± 0.7 min. iNAV CE-MRA quality score was higher (*p* < .001); average difference was 1.4 ± .08 at the sinus of Valsalva (SOV), 1.3 ± .08 at the sinotubular junction (STJ), and .87 ± .10 at the ascending aorta (AAO). Major/minor diameter interobserver agreement was better for iNAV CE-MRA (SOV ICC = .87–.93; STJ ICC = .95–.96; AAO ICC = .96–.97) vs. TR-MRA (SOV ICC = .69–.82; STJ ICC = .78–.83; AAO ICC = .89), as was intraobserver agreement (SOV ICC = .93–.95; STJ ICC = .94–.96; AAO ICC = .96–.97) vs. TR-MRA (SOV ICC = .81–.88; STJ ICC = .72–.73; AAO ICC = .87–.93).

**Conclusion:**

iNAV CE-MRA is feasible within a clinically reasonable scan time, provides superior image quality, and measurement reproducibility vs. TR-MRA.

## Background

The 2022 ACC/AHA Guideline for the Diagnosis and Management of Aortic Disease and 2024 ESC Guidelines for the Management of Peripheral Arterial and Aortic Diseases provide imaging recommendations, including the use of gated computed tomography (CT), or magnetic resonance angiography (MRA) for aortopathy assessment (1, 2). The carcinogenic risk associated with CT remains a controversial issue (3, 4); therefore, in younger patients and/or those in the need of serial imaging, MRA is a strong consideration (5). Although many institutions employ contrast-enhanced MRA without electrocardiogram (ECG) gating due to the challenge of triggering in the MR environment, ECG gating can be used with both contrast enhanced (CE) and non-contrast enhanced (non-CE) techniques to minimize cardiac motion artifacts and improve the reproducibility of proximal aortic diameter measurements (6). Although superior to gated first pass CE-MRA in terms of cardiac motion suppression (7), segmented acquisitions have historically required diaphragmatic navigator gating (dNAV) to provide respiratory motion compensation and prospective motion correction. However, scan inefficiency from low navigator acceptance rates (8) and residual motion artifacts from inaccurate slab tracking ratio result in suboptimal image quality, and an unpredictable scan duration. Furthermore, the limitations of non-CE techniques require tradeoffs between inhomogeneity artifacts inherent to the balanced steady-state free precession (bSSFP) readout; non-uniform fat suppression/risk of inadvertent water signal loss with frequency selective fat saturation (9); or the inefficiency of dual-echo gradient echo (Dixon T2 prep GRE) methods. Although the latter provides robust image quality in post-surgical patients, fat/water swaps (commonly in areas of turbulent flow) may be observed in up to 40% of exams (10, 11).

Inversion recovery gradient recall echo (IR GRE) after intravascular contrast agent administration shows significantly improved blood pool homogeneity, signal to noise (SNR), contrast to noise (CNR), and reduced artifacts from intrathoracic metal compared to T2 prep bSSFP non-CE MRA (12). Furthermore, the use of spectral fat saturation pre-pulses is avoided; echo/repetition times can be minimized, resulting in increased efficiency compared to Dixon techniques. Unfortunately, intravascular agents, such as ferumoxytol are incompatible with late gadolinium enhancement (LGE) imaging and bolus use (13), which sacrifices myocardial scar and dynamic MRA assessment respectively. Gadolinium based contrast agents (GBCA) have a shorter optimal intravascular imaging window for ECG gated free-breathing CE-MRA acquisition, which like non ECG gated techniques, makes this approach prone to mistiming errors related to contrast delivery. Time resolved MRA (TR-MRA) utilizes keyhole imaging with view sharing, phase partial Fourier, and parallel imaging to provide rapid multiphase acquisition with temporal resolution as short as 1–2 s, thus avoiding the aforementioned issue.

Recently, image-based navigators (iNAVs) have been implemented to provide 100% respiratory scan efficiency (14), and model-free respiratory motion correction, in conjunction with variable density spiral-like Cartesian trajectory sampling (VD-CASPR) for image acceleration. The use of such framework may overcome the use of a GBCA with weak albumin binding properties by significantly reducing CE-MRA acquisition duration. Recent iterations of iNAV directly track the signal of the blood pool. The effect of undiluted GBCA delivery and fluctuations in intravascular signal intensity over a short duration on iNAV tracking behavior may be deleterious (Supplementary Video 1). Resultant inaccurate estimations of respiratory motion could make iNAV CE-MRA of questionable value over standard non gated acquisitions.

We hypothesize that high fidelity, highly accelerated whole-chest gated CE-MRA using a research sequence with VD-CASPR and iNAV based non-rigid motion correction (iNAV CE-MRA) can be acquired within a clinically reasonable scan time. We also hypothesize that CE-MRA with an extracellular GBCA total dose of .15 mmol/kg is feasible using iNAV precision triggering (iNAV fluoro trigger) in conjunction with iNAV CE-MRA, while retaining dynamic information provided by TR-MRA.

Specifically, we aim to utilize the aforementioned framework (14) for up to 1.2 mm isotropic whole-chest MRA acquisition in 4–5 min; compare image quality, and intraobserver/interobserver variation of vessel measurements using the proposed method to TR-MRA.

## Materials and methods

### Research sample population

This was a single-center prospective study with enrollment conducted from 10/2022 to 3/2023. 31 consecutive patients undergoing clinical MRI for various indications were recruited for additional MRA imaging with another 9 (including 5 patients with known aortopathy) undergoing only thoracic MRA with TR-MRA and iNAV CE-MRA. Patients signed informed consent prior to enrollment. The sample size was based on previous substantiated literature comparisons between IR GRE and TR-MRA (15, 16). The study protocol was approved by the St Francis Hospital IRB, Roslyn, NY (reference # 21-06). HIPAA compliant research was performed in accordance with the Declaration of Helsinki.

### MRA image acquisition

All studies were performed on a 1.5 T Scanner (MAGNETOM Sola, Siemens Healthineers AG, Forchheim, Germany). The contrast injection protocol and inversion time selection were based on previous literature (17), which covered an acquisition duration of 4 min for pulmonary vein MRA. In order to adapt to an acquisition duration of 5 min for larger anatomical coverage, the continuous infusion rate was decreased from .20 ml/s to .16 ml/s. A total of .15 mmol/kg gadobutrol (Gadavist, Bayer pharmaceuticals) was used per patient.

Prior to TR-MRA, a test run of the iNAV CE-MRA research sequence was performed to ensure gradient noise amplitude did not interfere with vectocardiogram gating; to serve as a copy reference for saturation bands, slice prescription, and iNAV placement; and to provide automatic pre-scan tuning values (i.e., shimming), for the iNAV CE-MRA.

The diastolic rest period was determined using free breathing, four or three chamber bSSFP cine. For patients with irregular rhythms, or ventricular rates ≥80 BPM, the systolic rest period was selected. The data window duration was further adjusted from the default maximum value of 130 ms if needed to allow for a scan duration of under 5 min.

**Figure 1:**
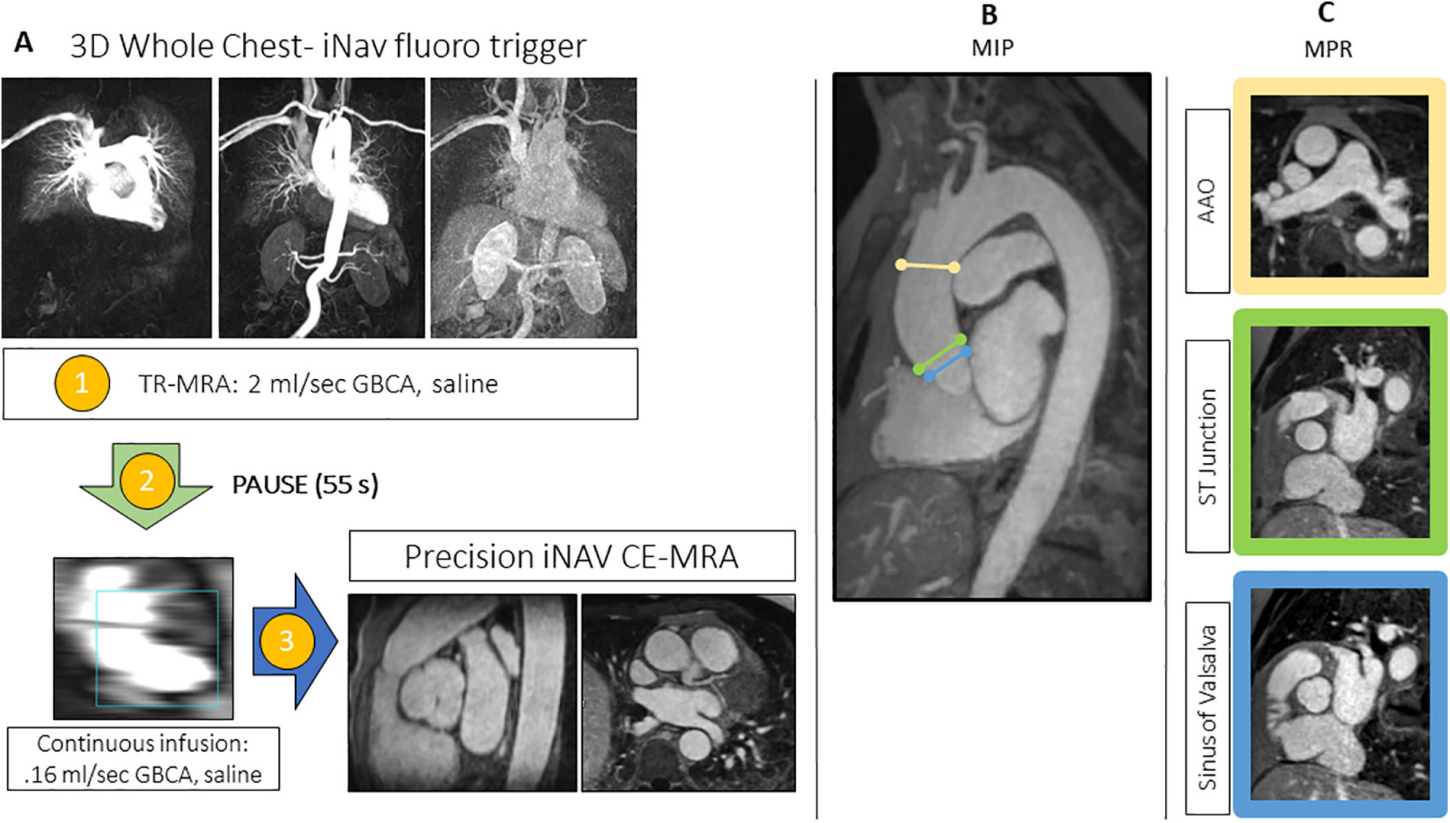
**(A)** The image-based navigator (iNAV) fluoro trigger method for whole-chest MR angiography. Two identical 3D whole heart program steps are created in the workflow. Following the time resolved (TR)-MRA (1), there is a pre-programed 55s injector pause (2). The first iNAV CE-MRA program step, which is used only to monitoring the passage of the continuous infusion, is started after TR-MRA reconstruction and patient instructions. The final step (3), which will run to completion, is triggered at the peak of the continuous infusion. **(B)** Overview MIPs of the landmarks for diameter measurements with representative MPR images **(C)** at the following levels: sinus of Valsalva, sinotubular (ST) junction, and ascending aorta (AAO) at the level of the pulmonary artery bifurcation.

TR-MRA with temporal resolution of 2.1 s, and nearly equivalent pixel area to the iNAV CE-MRA was performed (Figure 1) by injection of .05 mmol/kg of gadobutrol at 2 ml/s, and 20 ml of saline at the same rate (18). The saline injection was followed by a pre-programmed injector pause of 55 s, to account for the acquisition length of the TR-MRA (82 s), and image reconstruction. Afterwards, .1 mmol/kg of GBCA was infused at .16 ml/s, followed by 20 ml–30 ml of saline at the same rate (19).

**Figure 2:**
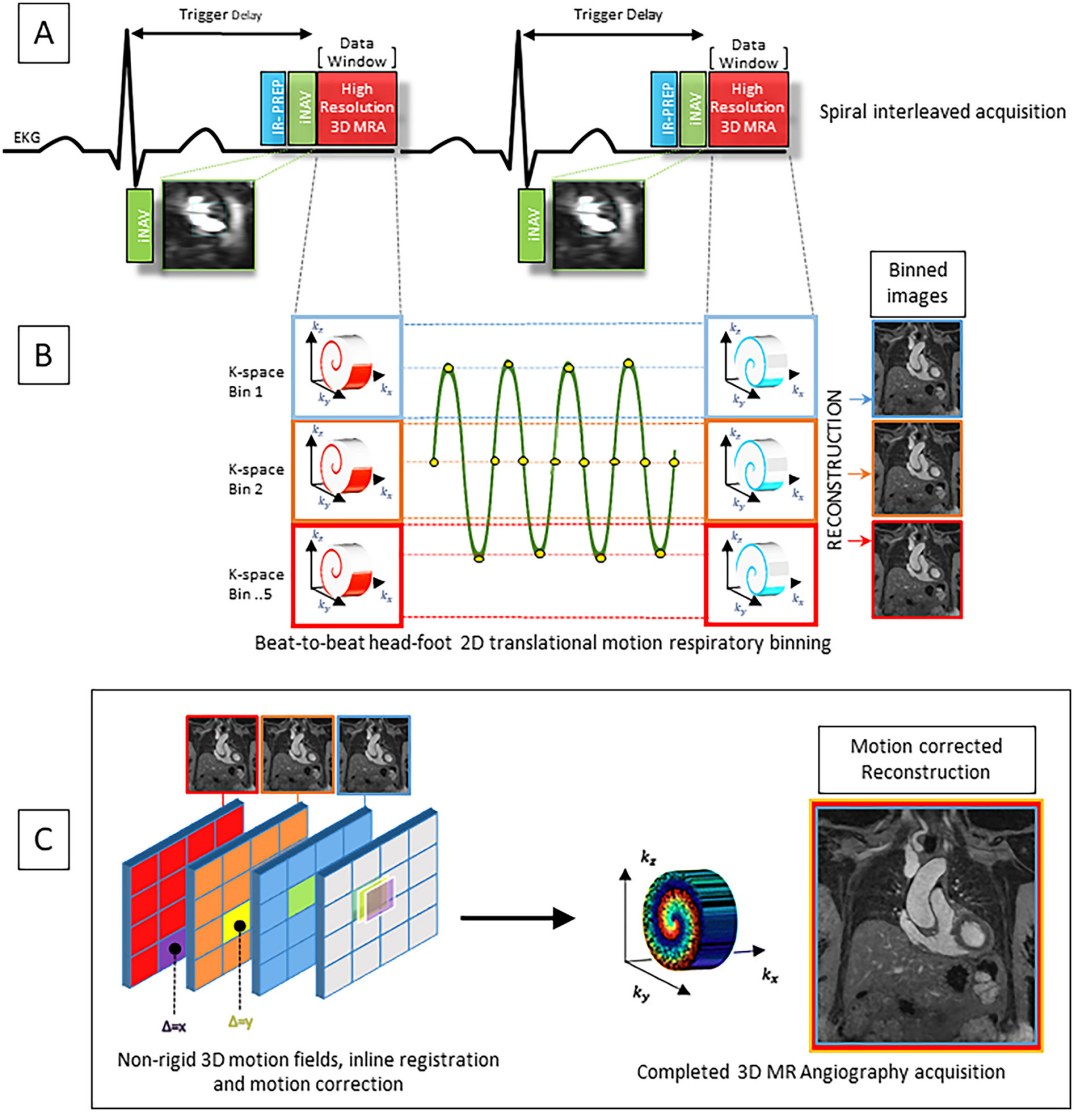
**(A)** Pulse sequence overview for the contrast enhanced MR angiography featuring VD-CASPR and inversion recovery (IR) gradient recall echo readout. K space data is acquired in an alternating spiral-like Cartesian interleaved trajectory and according to golden angle rotation. **(B)** Image-based navigator (iNAV) provides joint estimation of 2D translational respiratory motion in the head-foot and left-right directions. 5 equally populated k-space bins are subdivided based on 2D iNAV head-foot displacement data encompassing the entire respiratory cycle, allowing for 100% respiratory scan efficiency. **(C)** Images are reconstructed at each respiratory position. 3D non-rigid motion fields allow registration of reconstructed images to the reference end-expiratory position. The final completed iNAV CE-MRA acquisition is reconstructed using iterative SENSE with integrated inline motion correction.

Upon peak of the continuous infusion dose in the pulmonary artery, the final iNAV CE-MRA measurement was run to completion. Sequence parameters for the TR-MRA and iNAV CE-MRA are provided in Table 1; the pulse sequence overview for iNAV CE-MRA is provided in Figure 2. In 34 patients, iNAV CE-MRA was acquired at a spatial resolution of 1.31 × 1.31 × 1.57 mm (iNAV CE-MRA_standard_) with a nominal acceleration factor of 3.2. To test the feasibility of isotropic imaging in the remaining 6 patients, 1.2 mm isotropic (1.25 × 1.25 × 1.2 mm) spatial resolution with a nominal acceleration factor of 4.1 was used (iNAV CE-MRA_isotropic_). Both iNAV CE-MRA groups were reconstructed with iterative sensitivity encoding (SENSE).

**Table 1:** Sequence parameters for whole-chest time resolved (TR)-MRA and inversion recovery gradient recall echo (IR GRE) image-based navigator (iNAV) CE-MRA.

Imaging parameters for whole-chest TR-MRA and IR GRE MRA			
MRA sequence	TR-MRA	iNAV CE-MRA_Standard_ (VD-CASPR IR GRE)	iNAV CE-MRA_Isotropic_ (VD-CASPR IR GRE)
FOV (coronal)	400 × 300 × 160 mm	380 × 380 × 157 mm	360 × 360 × 153 mm
In-plane spatial resolution	1.13 × 1.62 mm	1.31 × 1.31 mm	1.25 × 1.25 mm
Phase oversampling	50%	30%	30%
Slice thickness	2 mm	1.4–1.5 mm	1.2 mm
Slice resolution	54%	90%	100%
Acceleration factor	GRAPPA 3	3.2	4.1
Sampling distribution	Elliptical scanning 15% (region A), 20% (region B)	Elliptical scanning	Elliptical scanning
Bandwidth	676 Hz/px	755 Hx/px	755 Hx/px
Flip angle	23°	18°	18°
Inversion time	N/A	200–240 ms (systolic) 290 ms (diastolic)	290 ms (diastolic)
TE/TR	.9 ms/2.4 ms	1.1 ms/3.34 ms	1.17 ms/3.44 ms
Data window duration	N/A	78–155 ms (23–46 segments)	127–161 ms (37–47 segments)

### Image analysis

Anonymized images were analyzed independently by 3 cardiologists with 4, 1, and 4 years of experience respectively [physicians 1 (RP), 2 (SB), and 3 (AL)] in a blinded fashion. Image quality at the sinus of Valsalva (SOV), sinotubular junction (STJ), and ascending aorta (AAO) segments (segmental image quality) was assessed by all three physicians according to the following scale: (1) non-diagnostic; (2) poor-significant blurring; (3) good-mild blurring; and (4) excellent.

Major/minor diameter measurements were made at 3 landmarks (Figure 1) by physicians 1 and 2. Using a randomized sample of 20 data sets, measurements were repeated at the level of the SOV, STJ, and AAO by physician 1 for intraobserver analysis.

### Statistical analysis

The mean difference and reliability of measurements and image quality was compared between readers and both sequences using paired *t*-tests and repeated-measures ANOVA as appropriate. The reproducibility of the segmental measurements were evaluated continuously and per score category through the use of the concordance correlation coefficient or Cohen's kappa as appropriate. Analyses were performed by JW using SAS version 9.4 (Cary, NC.), 2013. Where applicable, *P* values of <0.05 denotes statistical significance.

## Results

Scan time for all iNAV CE-MRA exams was 4.2 ± .7 min; 4.2 ± .7 min for iNAV CE-MRA_standard_; and 4.2 ± .6 min for iNAV CE-MRA_isotropic_. Heart rate was 64 ± 15 beats per minute during the iNAV CE-MRA image acquisition. Inline reconstruction time was 1:40 s. Demographics of the study population is provided in Table 2.

**Table 2:** Patient demographics for the study population.

Baseline demographics and medical history		
Variable	Mean, *N*	SD, %
Age	57	13.6
Female gender (*N*, %)	16	40%
BMI (kg/m^2^)	29.36	5.86
BSA (m^3^)	2	0.24
Heart rate at CMR (BPM)	66	11
Rhythm at time of MRI		
Sinus rhythm (*N*, %)	39	98%
Atrial fibrillation (*N*, %)	1	2%
Hypertension (*N*, %)	20	50%
Coronary artery or peripheral vascular disease (*N*, %)	11	28%
Diabetes (*N*, %)	3	7%
NYHA heart failure class ≥ 2 (*N*, %)	4	10%
Aortic aneurysmal disease ≥ 4cm	5	13%
History of aortic repair	3	8%
Aortic valve disease (stenosis, regurgitation) ≥mod (*N*, %)	1	2%
Valvular repair or replacement (*N*, %)	4	10%
Congenital heart disease (*N*, %)	3	8%

### Image quality

**Figure 3:**
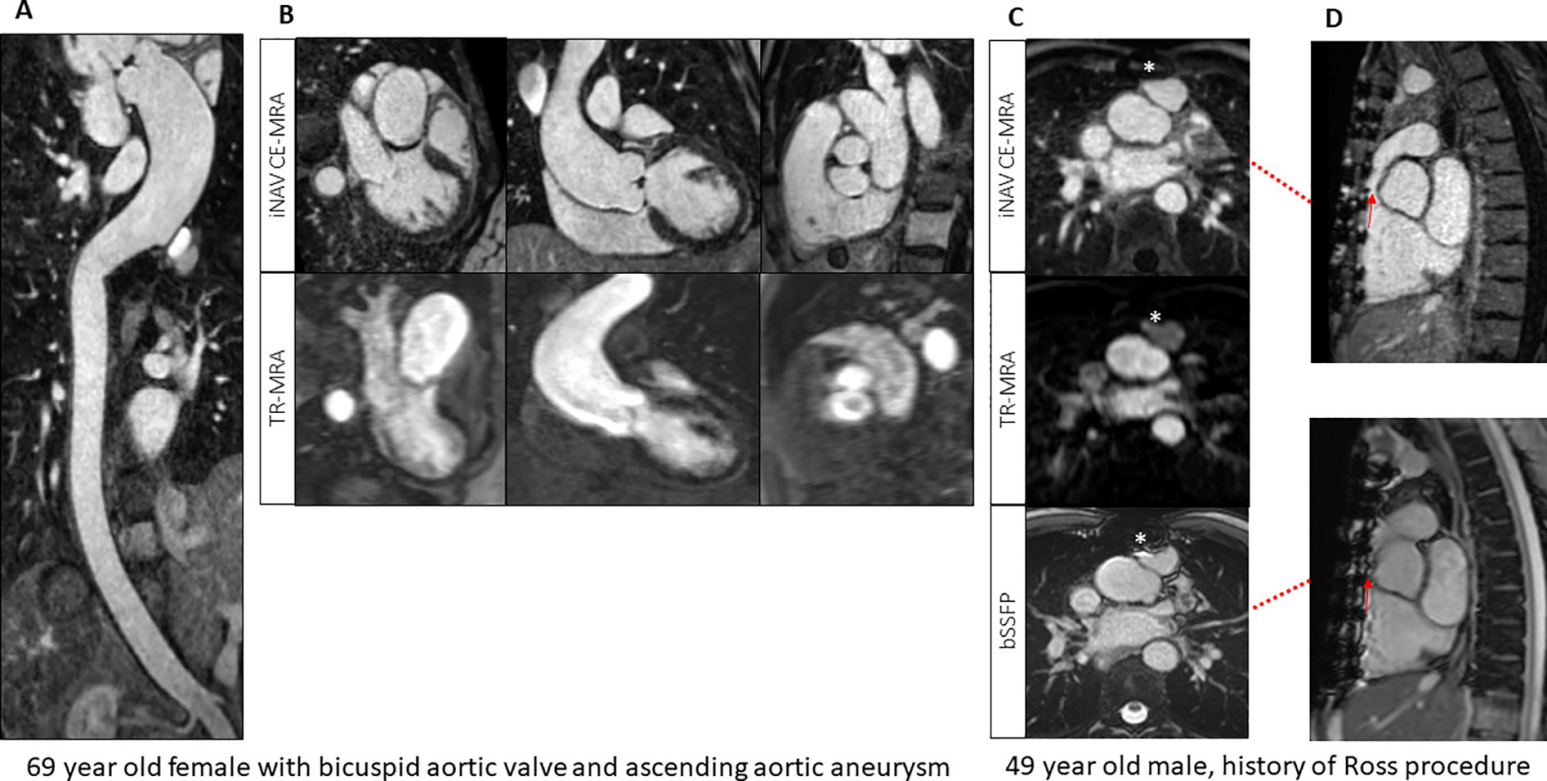
**(A)** Image-based navigator (iNAV) CE-MRA curved MPR from a patient with bicuspid aortic valve and aortopathy. **(B)** Comparison between time resolved (TR) and iNAV CE-MRA in the same patient. **(C,D)** Reduced metallic artifacts (*) from TR-MRA and iNAV CE-MRA compared to 2D balanced steady-state free precession (bSSFP) in a patient post Ross procedure.

**Figure 4:**
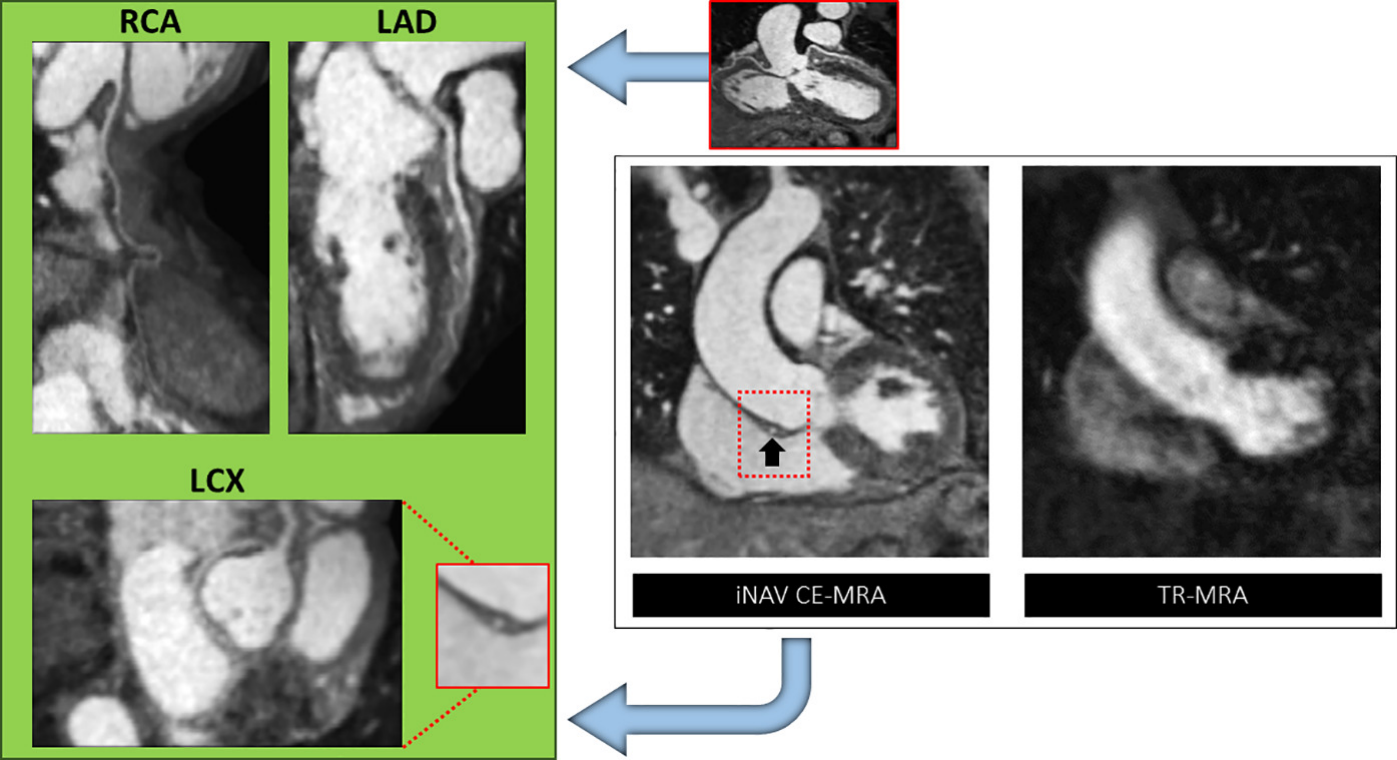
A case of anomalous left circumflex arising (LCX) from the right coronary artery (RCA) base. Right panel: The abnormality (black arrow) is not demonstrated on time resolved (TR)-MRA due to cardiac motion but clearly defined with gated image-based navigator (iNAV) CE-MRA. Left panel: MPR reformats of the RCA, left anterior descending (LAD) and LCX.

The mean image quality scores per segment for physicians are displayed in Table 3. For the 6 iNAV CE-MRA_isotropic_ exams, every segment (3 per patient, total 18) was scored as excellent by all physicians. A representative 1.2 mm isotropic acquisition is presented in Supplementary Figure 1. Pooled segmental iNAV CE-MRA image quality scores were significantly higher than TR-MRA (*p* < .001) for all levels with an average difference of 1.4 ± .08 for SOV, 1.3 ± .08 for STJ, and.87 ± .10 for AAO respectively. Two direct comparison examples of TR-MRA vs. iNAV CE-MRA quality are shown in Figures 3, 4 respectively.

**Table 3:** Interobserver variation for time resolved (TR)-MRA and image based navigator (iNAV) CE-MRA segmental measurements (in cm) and image quality.

Interobserver variation and image quality													
Measurement level		TR-MRA: Physician 1—Physician 2			Image quality^a^			iNAV CE-MRA: Physician 1—physician 2			Image quality^a^		
		Vessel diameter mean difference	*P* value	ICC (95% LOA)	Mean IQ score Physician 1	Mean IQ score Physician 2	Mean IQ score Physician 3	Vessel diameter mean difference	*P* value	ICC (95% LOA)	Mean IQ score physician 1	Mean IQ score physician 2	Mean IQ score physician 3
Sinus of Valsalva	Major	0.11 ± 0.25	0.012*	0.82 (0.68, 0.90)	2.83 ± 0.59	2.25 ± 0.54	2.53 ± 1.04	−0.092 ± 0.12	<0.001*	0.93 (0.88, 0.96)	3.95 ± 0.22*	4 ± 0*	3.73 ± 0.55*
	Minor	0.098 ± 0.39	0.117	0.69 (0.48, 0.82)				−0.028 ± 0.24	0.47	0.87 (0.77, 0.93)			
Sinotubular junction	Major	0.068 ± 0.23	0.068	0.83 (0.71, 0.91)	2.78 ± 0.48	2.25 ± 0.54	2.6 ± 1.03	0.029 ± 0.13	0.17	0.95 (0.90, 0.97)	3.93 ± 0.27*	4 ± 0*	3.73 ± 0.55*
	Minor	0.027 ± 0.26	0.52	0.78 (0.61, 0.88)				0.028 ± 0.1	0.094	0.96 (0.93, 0.98)			
Ascending aorta	Major	0.039 ± 0.25	0.34	0.89 (0.81, 0.94)	2.90 ± 1.15	2.88 ± 0.72	3.25 ± 0.87	0.004 ± 0.15	0.89	0.96 (0.93, 0.98)	3.95 ± 0.22*	4 ± 0*	3.7 ± 0.56*

* Notes statistical significance at the *p* < .05 level.

^a^ Image quality scale: (1) non-diagnostic; (2) poor-significant blurring; (3) good-mild blurring; and (4) excellent.

### Interobserver agreement analysis

**Figure 5:**
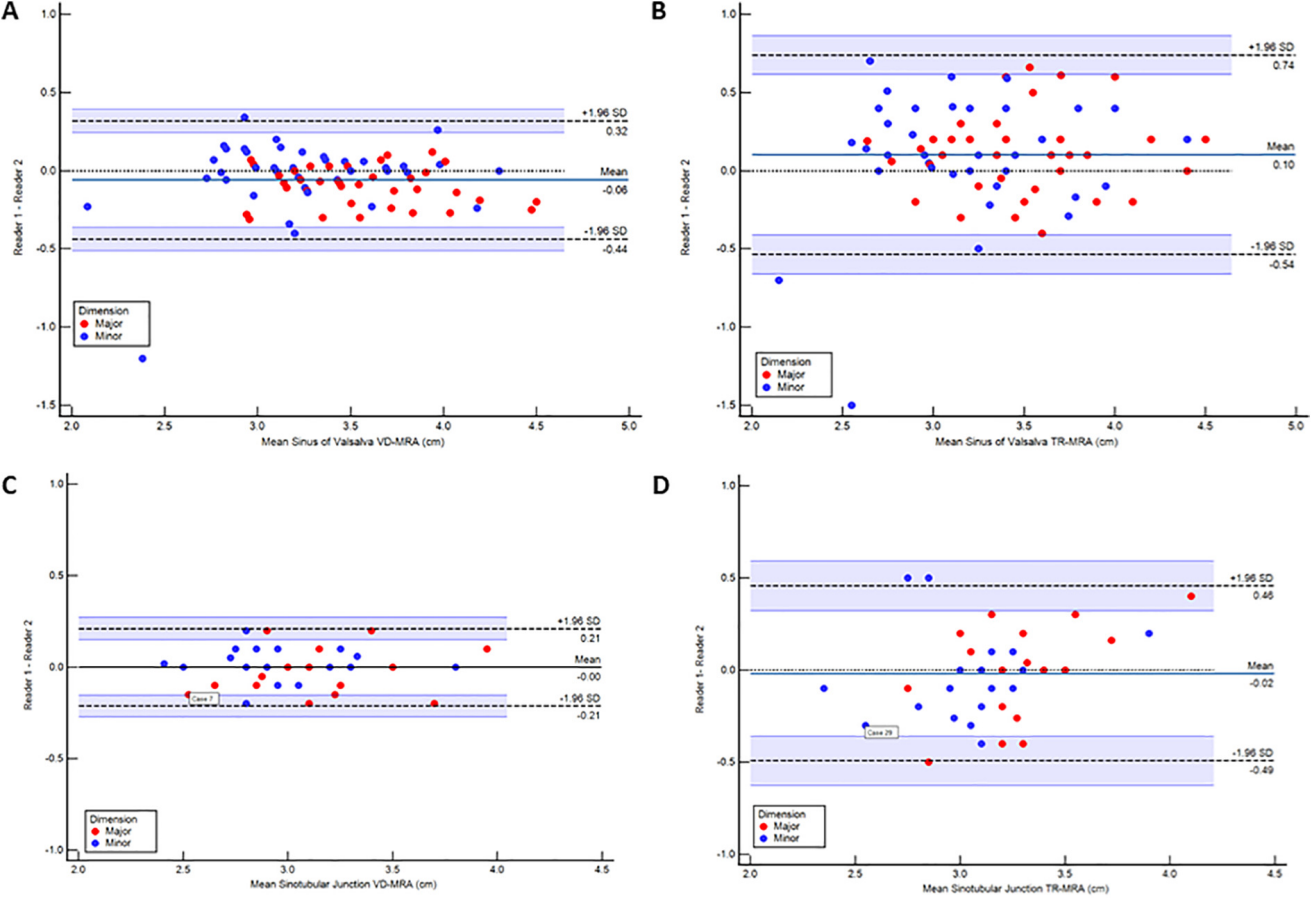
Bland-Altman plots demonstrating interobserver agreement between readers measuring at the level of the sinus of valsalva using image-based navigator (iNAV) CE-MRA **(A)** and time resolved (TR)-MRA **(B)** as well as intraobserver agreement within readers measuring at the level of the sinotubular junction using iNAV CE-MRA **(C)** and TR-MRA **(D)** All measurements are reported in cm.

The interobserver mean difference between major and minor diameter measurements at each level between physician 1 and physician 2 is reported in Table 3. SOV major diameter mean difference for iNAV CE-MRA and TR-MRA was statistically significantly different between observers; however, these differences (.92 mm and 1.1 mm respectively) are unlikely to be clinically significant. Measurement agreement between physicians for iNAV CE-MRA was excellent at every level, with intraclass correlation coefficient (ICC) values ranging from .87 to .97. For TR-MRA measurement agreement was good to excellent, with ICC values ranging from .69 to .82. The lowest overall agreement (major and minor diameter agreement averaged) was observed at the level of SOV for TR-MRA (SOV_Major_ ICC = .69, SOV_Minor_ ICC = .82). A Bland-Altman plot of the major and minor diameters for interobserver agreement at this level is outlined in Figures 5a,b. Additionally, the second and third lowest interobserver ICC values were for TR-MRA at the STJ and AAO levels respectively. Bland-Altman interobserver plots for both iNAV CE-MRA and TR-MRA at the STJ and AAO levels can be found in Supplementary Figure 2.

### Intraobserver agreement analysis

Agreement between both measurements for iNAV CE-MRA was excellent, with ICC values ranging from .93 to .97 (Table 4). For TR-MRA, ICC values ranged between .72 and .93 consistent with good to excellent agreement. The intraoberver mean diameter difference was not statistically significant for TR-MRA or iNAV CE-MRA at any level. The lowest overall agreement between measurements (major and minor diameter agreement averaged) was seen at the levels of the SOV (SOV_Major_ ICC = .81, SOV_Minor_ ICC = .88), STJ (STJ_Major_ ICC = .72, STJ_Minor_ ICC = .73), and AAO (AAO_Major_ ICC = .87, AAO_Minor_ ICC = .93)- all for TR-MRA. A Bland-Altman plot of the major and minor diameters for intraobserver agreement at the STJ level (lowest averaged agreement for TR-MRA) is demonstrated in Figures 5c,d. Bland-Altman intraobserver plots for both iNAV CE-MRA and TR-MRA at the remaining 2 levels can be found in Supplementary Figure 3.

**Table 4:** Intraobserver variation of segmental measurements (in cm) for physician 1.

Intraobserver variation: measurement 1 vs. measurement 2							
Measurement level		Physician 1: Measurement 1- Measurement 2					
		iNAV CE-MRA diameter mean difference	*P* value	ICC (95% LOA)	TR-MRA diameter mean difference	*P* value	ICC (95% LOA)
Sinus of valsalva	Major	0.015 ± 0.11	0.56	0.95 (0.88, 0.98)	−0.012 ± 0.22	0.82	0.81 (0.59, 0.92)
	Minor	−0.056 ± 0.17	0.16	0.93 (0.82, 0.97)	−0.065 ± 0.2	0.16	0.88 (0.72, 0.95)
Sinotubular junction	Major	−0.018 ± 0.12	0.53	0.94 (0.85, 0.98)	−0.008 ± 0.25	0.89	0.72 (0.43, 0.87)
	Minor	0.017 ± 0.09	0.43	0.96 (0.89, 0.98)	−0.028 ± 0.24	0.61	0.73 (0.42, 0.89)
Ascending aorta	Major	0.039 ± 0.15	0.26	0.96 (0.89, 0.98)	−0.069 ± 0.28	0.28	0.87 (0.7, 0.95)
	Minor	0.048 ± 0.14	0.14	0.97 (0.91, 0.99)	−0.091 ± 0.2	0.052	0.93 (0.83, 0.97)

## Discussion

In the current study, we achieve whole-chest CE-MRA up to 1.2 mm isotropic spatial resolution in under 4.5 min using iNAV and VD-CASPR framework with a motion-compensated iterative SENSE reconstruction. An average of good or excellent image quality was achieved in all patients at every segment. Reconstruction times were also clinically acceptable, completed in under 2 min on the scanner GPU. iNAV mistracking behaviors were not evident.

In comparison to TR-MRA, image quality, interobserver, and intraobserver agreement were significantly better. Our results are similar to Naehle et al. (15), Naehle et al. (20), and Dabir et al. (16) who also used dynamic ungated CE-MRA for the comparison group.

The ability to perform high resolution, efficient gated CE-MRA is desirable. First pass, breath held MRA with adaptable ECG triggering, flexible k-space segmentation, and elliptical scanning was previously used to reduce motion in the aortic root and ascending aorta (21). The time to center per heart beat could be assigned to the diastolic quiescent period, resulting in reduced cardiac motion. Elliptical scanning provided a small reduction of scan time. Furthermore, early and late cardiac cycles could be accounted for by sampling the periphery of k-space during periods where the trigger was expected, but not received. Using a minimal trigger delay, scan times approached that of ungated first pass CE-MRA. Despite the advantage in acquisition time and ease of use, suppression of cardiac motion in the aortic root was inferior to dNAV respiratory gating (7).

Likewise, dNAV gating, which has historically been paired with IR GRE MRA and conventional parallel imaging has its own inherent limitations, the most notable being scan inefficiency, residual respiratory motion, and chest wall ghosting due to use of a presumptuous slab tracking ratio. Compensatory adjustments for the former limitation have included the use of double dose (.2 mmol/kg) extracellular GBCA, imaging at higher field strengths, dilution of contrast, and/or reduced spatial resolution (16, 22, 23).

3D radial self-navigated sequences are also another potentially viable method for high quality, high resolution IR GRE MRA. Although vessel sharpness is preserved, SNR and CNR appear to be significantly higher using dNAV gating in a limited sample of patients (24). Using VD-CASPR sampling and 100% efficient iNAV motion correction to reduce scan time; and iNAV precision fluoro triggering, we are able to demonstrate the feasibility of 25% GBCA dose reduction compared to dNAV approaches (16, 22, 23), achieve 1.2 mm isotropic spatial resolution at 1.5 T, while not sacrificing the dynamic information of TR-MRA. The implications of this research include the development of high throughput gated CE-MRA particularly in post-surgical populations where suppression of susceptibility artifacts from the GRE readout are of value. The benefit of motion suppression from cardiac gating serves to increase reproducibility of vessel measurements and has implications for serial examinations used for long-term follow up (25).

## Limitations

There are limitations to the current study. Only 8% of the study population had prior aortic surgery; hence, the performance of the investigated MRA technique is unknown in these patients with inherent challenges to MRI. For our comparison, we used dynamic MRA similar to Naehle et al. (15), Naehle et al. (20), and Dabir et al. (16); ungated MRA is still commonly used in clinical practice. As such, we were not able to match spatial resolution between the two sequences. However, using TR-MRA enabled us to fit additional sequences for the purpose of research within the allocated clinical exam time, whereas gated non contrast MRA would not fit within the clinical workflow. Comparison with a gated MRA method should be the subject of future research. We did not explore the use of half molar GBCA (such as gadoterate). We did not explore alternative placements of the iNAV such as incorporating the blood pool of the ascending aorta (26). As respiratory motion of the aorta is translational, the traditional iNAV placement incorporating the left ventricular blood pool was thought to be sufficient. It is also possible that the 55 s injector pause can be reduced. Eliminating the injector pause may lead to minor peripheral or central venous enhancement at the end of the TR-MRA acquisition and potentially missing the initial arrival of the continuous infusion (in smaller patients or those with more robust cardiovascular function). However, iNAV CE-MRA contrast enhancement may be greater since GBCA is delivered over a shorter total duration. iNAV CE-MRA was also performed without spectral fat saturation, which carries the tradeoff between inadvertent water saturation and improved signal suppression from adipose tissue (15). Finally, respiratory binning quality may not be adequate in the context of erratic respirations leading to inaccurate motion estimation. Future work is needed to address the extremes of respiratory deviation encountered with problematic breathing patterns. iNAV CE-MRA overall image quality can also be degraded by irregular cardiac cycles. Arrhythmia rejection may further improve image quality in these instances, and could be the emphasis of further research.

## Conclusion

In summary, highly accelerated, high quality, high resolution whole-chest CE-MRA acquired within a clinically reasonable scan time is feasible using the proposed method, and provides superior image quality and reproducibility of vessel measurements compared to TR-MRA.

## Data Availability

The raw data supporting the conclusions of this article will be made available upon request to the corresponding author.

## Supplementary material

Supplementary Video S1 Failure of image-based navigator tracking (blue box) due to rapid signal intensity variation in the blood pool.

## References

[1] Isselbacher EM Preventza O Hamilton Black J Augoustides JG Beck AW Bolen MA 2022 ACC/AHA guideline for the diagnosis and management of aortic disease: a report of the American Heart Association/American College of Cardiology joint committee on clinical practice guidelines. Circulation. (2022) 146(24):e334–482. 10.1161/CIR.0000000000001106 36322642 PMC9876736

[2] Mazzolai L Teixido-Tura G Lanzi S Boc V Bossone E Brodmann M 2024 ESC guidelines for the management of peripheral arterial and aortic diseases. Eur Heart J. (2024) 45(36):3538–700. 10.1093/eurheartj/ehae179 39210722

[3] Amis ES Jr Butler PF. ACR white paper on radiation dose in medicine: three years later. J Am Coll Radiol. (2010) 7(11):865–70. 10.1016/j.jacr.2010.04.006 21040868

[4] Brenner DJ Hall EJ. Computed tomography–an increasing source of radiation exposure. N Engl J Med. (2007) 357(22):2277–84. 10.1056/NEJMra072149 18046031

[5] Gunn AJ Kalva SP Majdalany BS Craft J Eldrup-Jorgensen J Ferencik M ACR appropriateness criteria® nontraumatic aortic disease. J Am Coll Radiol. (2021) 18(5s):S106–18. 10.1016/j.jacr.2021.02.004 33958105

[6] van Kesteren F Elattar MA van Lienden KP Baan J Jr Marquering HA Planken RN. Non-contrast enhanced navigator-gated balanced steady state free precession magnetic resonance angiography as a preferred magnetic resonance technique for assessment of the thoracic aorta. Clin Radiol. (2017) 72(8):695.e1–6. 10.1016/j.crad.2017.03.003 28388971

[7] von Knobelsdorff-Brenkenhoff F Gruettner H Trauzeddel RF Greiser A Schulz-Menger J. Comparison of native high-resolution 3D and contrast-enhanced MR angiography for assessing the thoracic aorta. Eur Heart J Cardiovasc Imaging. (2014) 15(6):651–8. 10.1093/ehjci/jet263 24399340

[8] Correa Londono M Trussardi N Obmann VC Piccini D Ith M von Tengg-Kobligk H Radial self-navigated native magnetic resonance angiography in comparison to navigator-gated contrast-enhanced MRA of the entire thoracic aorta in an aortic patient collective. J Cardiovasc Magn Reson. (2021) 23(1):94. 10.1186/s12968-021-00774-9 34247640 PMC8274024

[9] Bley TA Wieben O François CJ Brittain JH Reeder SB. Fat and water magnetic resonance imaging. J Magn Reson Imaging. (2010) 31(1):4–18. 10.1002/jmri.21895 20027567

[10] Gietzen C Janssen JP Tristram J Cagman B Kaya K Terzis R Assessment of the thoracic aorta after aortic root replacement and/or ascending aortic surgery using 3D relaxation-enhanced angiography without contrast and triggering. Front Cardiovasc Med. (2025) 12:1532661. 10.3389/fcvm.2025.1532661 40144927 PMC11937005

[11] Pennig L Wagner A Weiss K Lennartz S Huntgeburth M Hickethier T Comparison of a novel compressed SENSE accelerated 3D modified relaxation-enhanced angiography without contrast and triggering with CE-MRA in imaging of the thoracic aorta. Int J Cardiovasc Imaging. (2021) 37(1):315–29. 10.1007/s10554-020-01979-2 32852711 PMC7878228

[12] Tandon A Hashemi S Parks WJ Kelleman MS Sallee D Slesnick TC. Improved high-resolution pediatric vascular cardiovascular magnetic resonance with gadofosveset-enhanced 3D respiratory navigated, inversion recovery prepared gradient echo readout imaging compared to 3D balanced steady-state free precession readout imaging. J Cardiovasc Magn Reson. (2016) 18(1):74. 10.1186/s12968-016-0296-4 27802802 PMC5090984

[13] FDA Drug Safety Communication. Safety announcement: FDA strengthens warnings and changes prescribing instructions to decrease the risk of serious allergic reactions with anemia drug feraheme (ferumoxytol). (2015).

[14] Bustin A Ginami G Cruz G Correia T Ismail TF Rashid I Five-minute whole-heart coronary MRA with sub-millimeter isotropic resolution, 100% respiratory scan efficiency, and 3D-PROST reconstruction. Magn Reson Med. (2019) 81(1):102–15. 10.1002/mrm.27354 30058252 PMC6617822

[15] Naehle CP Müller A Willinek WA Meyer C Hestermann T Gieseke J First-pass and steady-state magnetic resonance angiography of the thoracic vasculature using gadofosveset trisodium. J Magn Reson Imaging. (2009) 30(4):809–16. 10.1002/jmri.21919 19787726

[16] Dabir D Naehle CP Clauberg R Gieseke J Schild HH Thomas D. High-resolution motion compensated MRA in patients with congenital heart disease using extracellular contrast agent at 3 tesla. J Cardiovasc Magn Reson. (2012) 14(1):75. 10.1186/1532-429X-14-75 23107424 PMC3552711

[17] Craft J Weber J Li Y Cheng JY Diaz N Kunze KP Inversion recovery and saturation recovery pulmonary vein MR angiography using an image based navigator fluoro trigger and variable-density 3D cartesian sampling with spiral-like order. Int J Cardiovasc Imaging. (2024) 40(6):1363–76. 10.1007/s10554-024-03111-0 38676848

[18] Kramer CM Barkhausen J Bucciarelli-Ducci C Flamm SD Kim RJ Nagel E. Standardized cardiovascular magnetic resonance imaging (CMR) protocols: 2020 update. J Cardiovasc Magn Reson. (2020) 22(1):17. 10.1186/s12968-020-00607-1 32089132 PMC7038611

[19] Moaddab A Craft J Alshebani Y Biso S Schmidt M Kunze K Semi-automated measurements of the thoracic aorta assessed by cardiac imaging fellows: comparison with manual expert analysis. J Cardiovasc Magn Reson. (2024) 26(Supplement 1):100293. 10.1016/j.jocmr.2024.100293

[20] Naehle CP Kaestner M Müller A Willinek WW Gieseke J Schild HH First-pass and steady-state MR angiography of thoracic vasculature in children and adolescents. JACC Cardiovasc Imaging. (2010) 3(5):504–13. 10.1016/j.jcmg.2009.12.015 20466346

[21] Natsuaki Y Kroeker R Laub G Schmitt P Fin J. Advancements in the ECG-gated contrast-enhanced MR Angiography. Magnetom FLASH: Siemens Healthineers (2013). p. 32–8. Available at: https://mri-q.com/uploads/3/4/5/7/34572113/natsuaki_siemens_ecg_gated.pdf (Accessed April 24, 2025).

[22] Tandon A James L Henningsson M Botnar RM Potersnak A Greil GF A clinical combined gadobutrol bolus and slow infusion protocol enabling angiography, inversion recovery whole heart, and late gadolinium enhancement imaging in a single study. J Cardiovasc Magn Reson. (2016) 18(1):66. 10.1186/s12968-016-0285-7 27716273 PMC5052797

[23] Lam CZ Pagano JJ Gill N Vidarsson L de la Mora R Seed M Dual phase infusion with bolus tracking: technical innovation for cardiac and respiratory navigated magnetic resonance angiography using extracellular contrast. Pediatr Radiol. (2019) 49(3):399–406. 10.1007/s00247-018-4293-7 30443668

[24] Slesnick T McNeal GR Tandon A Sallee D Parks JW Zenge MO 3D contrast enhanced self navigated inversion recovery gradient echo coronary imaging in pediatric patients. J Cardiovasc Magn Reson. (2015) 17(Suppl 1):Q98. 10.1186/1532-429x-17-s1-q98

[25] Smith LR Darty SN Jenista ER Gamoneda GL Wendell DC Azevedo CF ECG-gated MR angiography provides better reproducibility for standard aortic measurements. Eur Radiol. (2021) 31(7):5087–95. 10.1007/s00330-020-07408-1 33409772

[26] Fotaki A Munoz C Emanuel Y Hua A Bosio F Kunze KP Efficient non-contrast enhanced 3D cartesian cardiovascular magnetic resonance angiography of the thoracic aorta in 3 min. J Cardiovasc Magn Reson. (2022) 24(1):5. 10.1186/s12968-021-00839-9 35000609 PMC8744314

