# Supplementary material for: Isotropic, high-resolution, whole-chest inversion recovery contrast-enhanced magnetic resonance angiography in under 4.5 min using image-based navigator fluoro trigger
Source: Front Cardiovasc Med. 2025 Apr 30;12:1549275. doi: 10.3389/fcvm.2025.1549275 (PMC12075228; doi:10.3389/fcvm.2025.1549275)
Supplement: Supplementary file 2 [file Datasheet1.pdf]

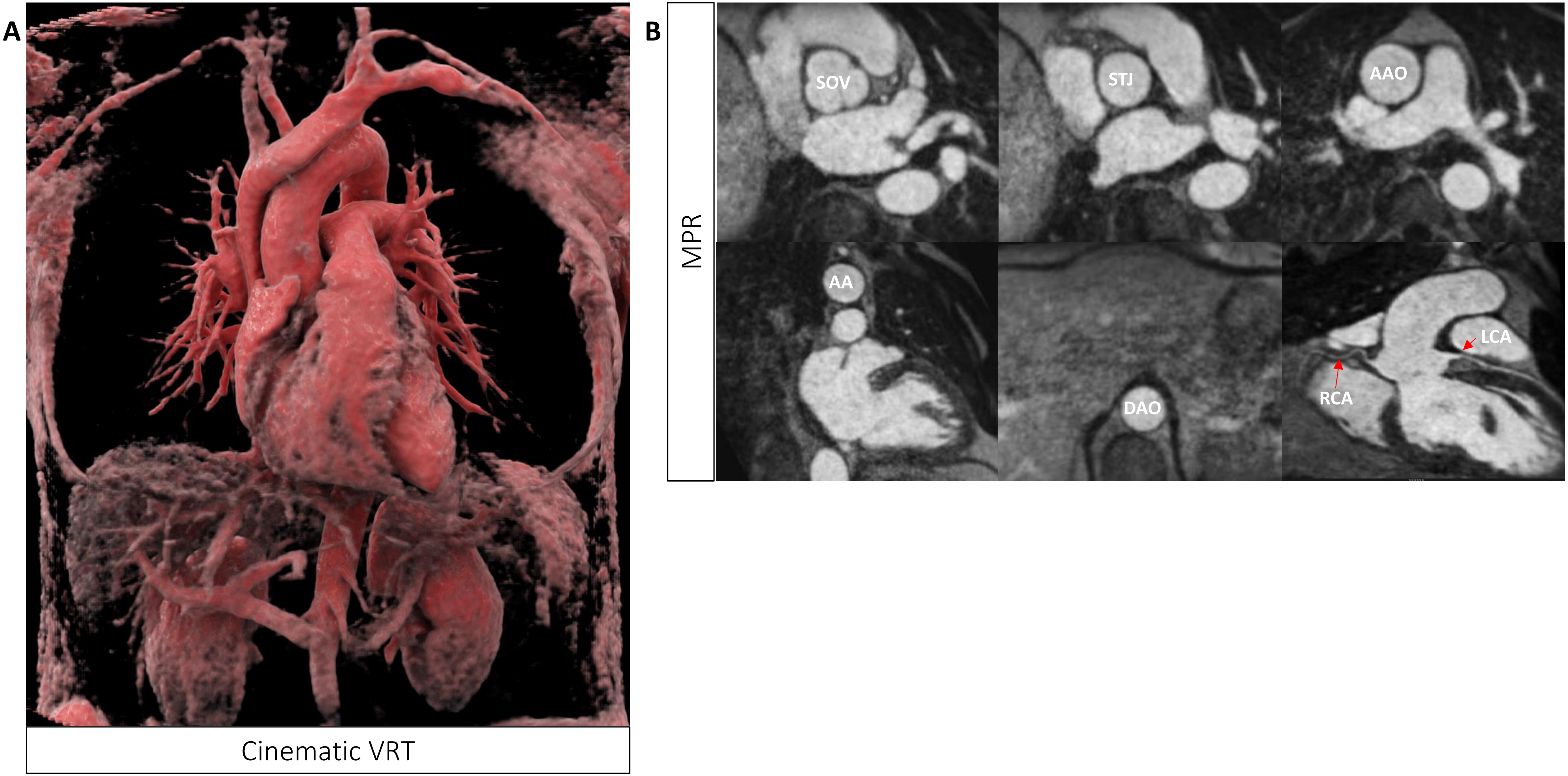

Supplementary figure 1: **(A)** Cinematic VRT and **(B)** Representative MPR images from isotropic 1.2 mm datasets. SOV: sinus of Valsalva; STJ: sinotubular junction; AAO: ascending aorta; AA: aortic arch; DAO: descending aorta; LCA: left coronary artery; RCA: right coronary artery.

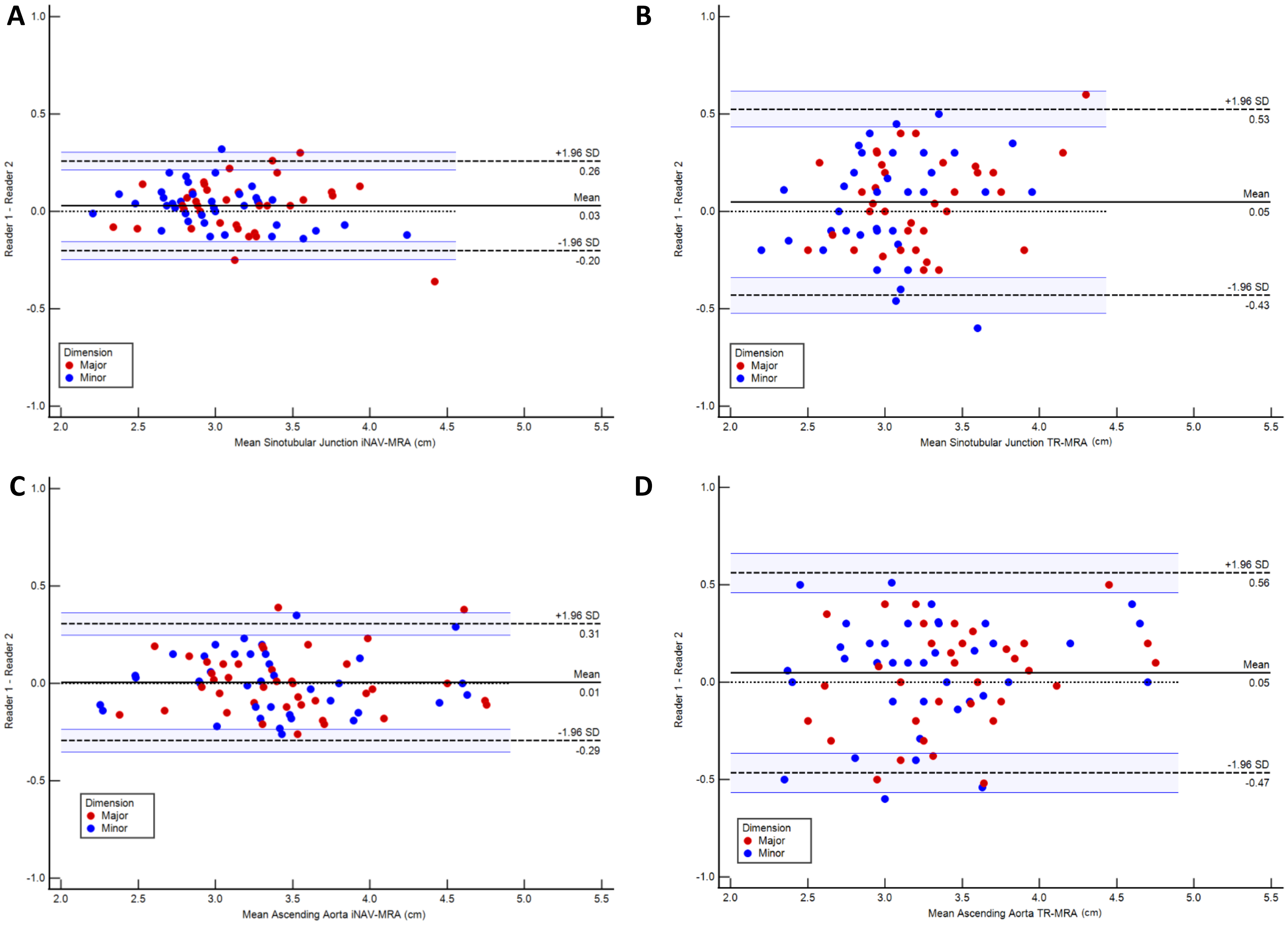

Supplementary figure 2: Bland-Altman plot of the sinotubular junction major and minor interobserver diameter measurements for iNAV CE-MRA **(A)**, and TR-MRA **(B)**; Bland-Altman plot of the ascending aorta major and minor interobserver diameter measurement for iNAV CE-MRA **(C)**, and TR-MRA **(D)**.

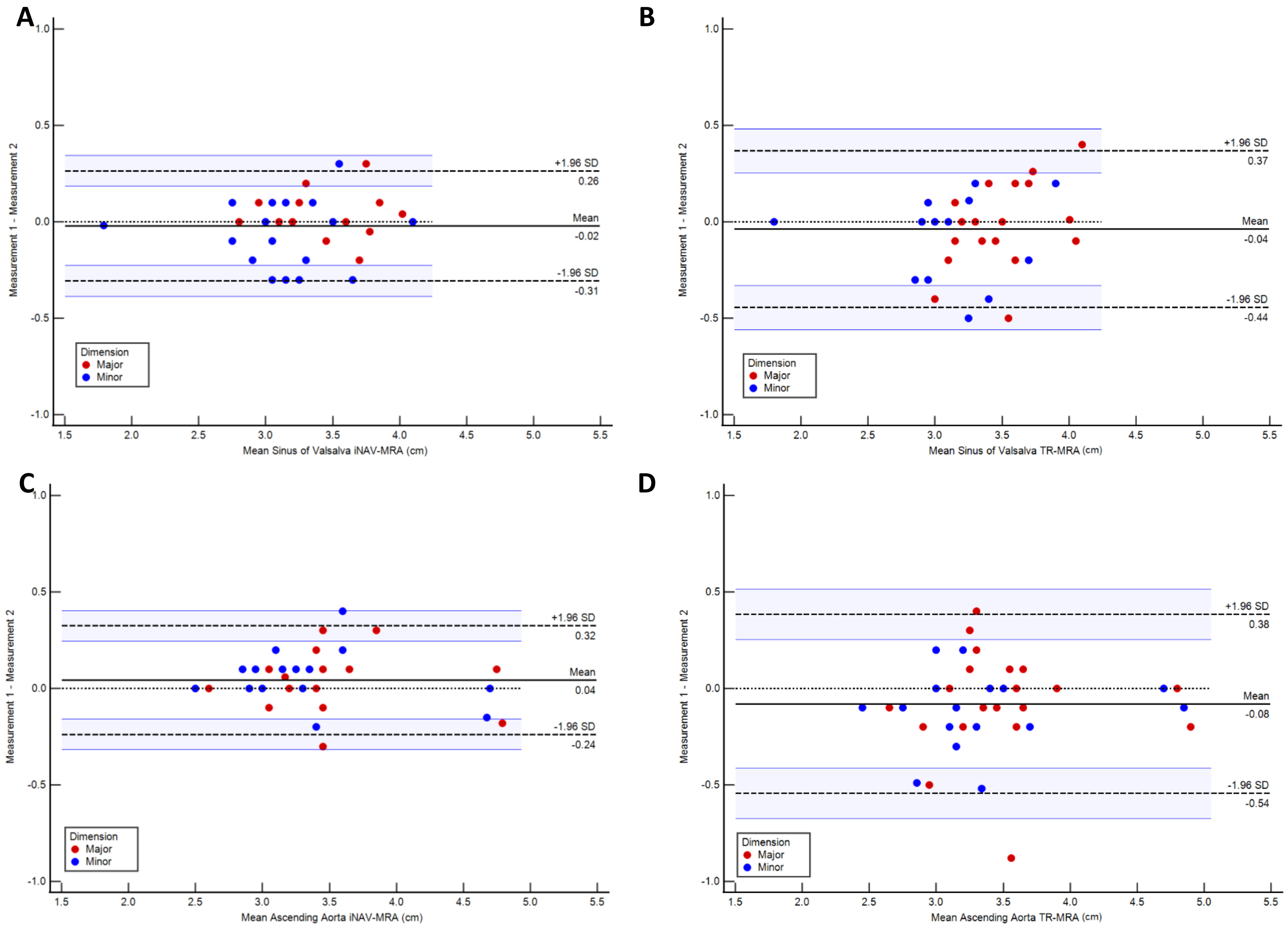

Supplementary figure 3: Bland-Altman plot of the sinus of Valsalva major and minor intraobserver diameter measurements for iNAV CE-MRA **(A)**, and TR-MRA **(B)**; Bland-Altman plot of the ascending aorta major and minor intraobserver diameter measurement for iNAV CE-MRA **(C)**, and TR-MRA **(D)**.
